# Supplementary material for: Role of the antiparallel double-stranded filament form of FtsA in activating the Escherichia coli divisome
Source: bioRxiv. 2024 Jun 30:2024.06.24.600433. Preprint. [Version 2] doi: 10.1101/2024.06.24.600433 (PMC11230281; doi:10.1101/2024.06.24.600433)
Supplement: Supplement 1 [file media-1.pdf]

Supplemental Figures and Tables

Role of the antiparallel double-stranded filament form of FtsA in activating the *Escherichia coli* divisome

Abbigale Perkins, Mwidy Sava Mounange-Badimi[1], and William Margolin[1]

Contents:

Figures – Fig. S1-S3 with legends

Tables – Tables S1-S2

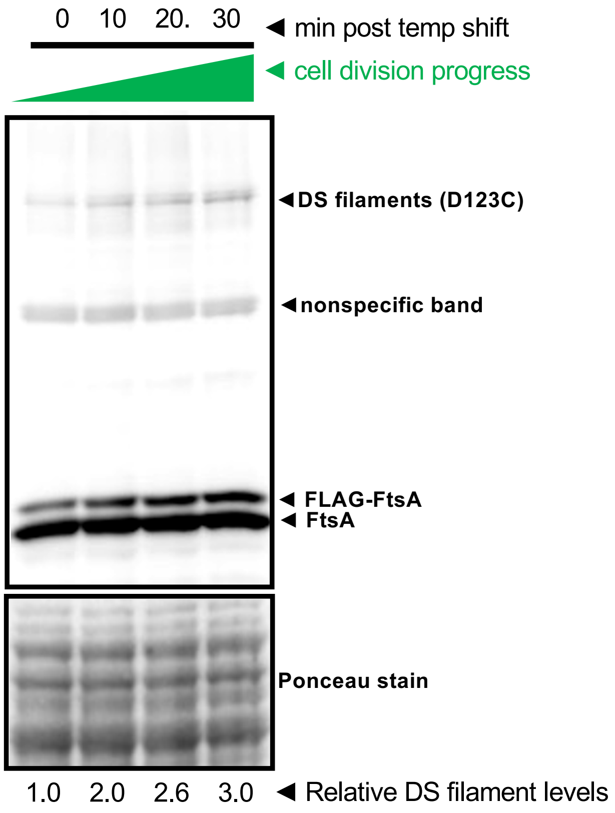

Fig. S1. DS filaments measured by crosslinking at D123C increase later in cell division. FtsA^{D123C} in *ftsZ84*(ts) (WM7612) was used instead of FtsA^{Q155C} to assay for DS filaments through *in vivo* crosslinking, probing immunoblots with anti-FtsA antibody, using the protocol described in Fig. 2B. As the distance between D123C residues in opposing antiparallel filaments is 3.7 Å (vs. 12.6 Å for Q155C), the shorter 5 Å dBBR crosslinker was used. A section of the same blot stained with Ponceau is shown below. Relative DS filament band intensities (ratio of crosslinked band to Ponceau stained protein at each time point, normalized to time zero) are shown at the bottom.

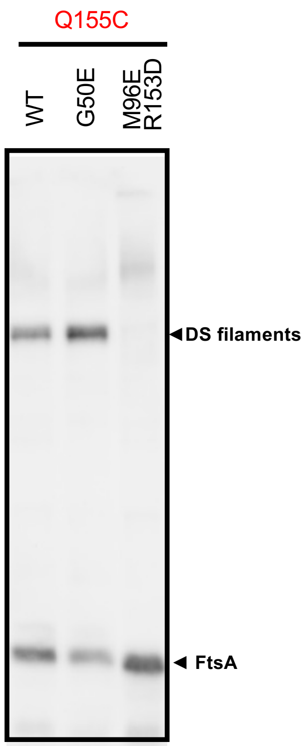

Fig. S2. FtsA$^{M96E\ R153D}$ also fails to form DS filaments in an *ftsL strain background.** WT, G50E or M96E R153D variants of FtsA also containing the Q155C residue for cysteine crosslinking (pWM7049, pWM7235, pWM7415) were each introduced into the *ftsL** WM4953 strain. After expression of each FtsA variant and crosslinking the Q155C residues with BMH, samples were separated by SDS-PAGE and probed with anti-FtsA on immunoblots.

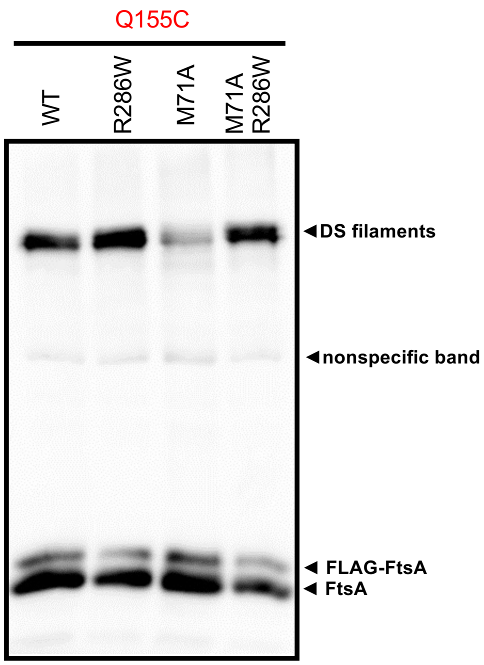

Fig. S3. The previously characterized dominant negative variant FtsA^{M71A} is deficient in forming DS filaments but can be rescued by R286W. The FtsA variants expressed from pDSW210-FLAG shown (expressed from pWM7150, pWM7239, pWM7613, pWM7614, all in WM1074) were grown according to the standard crosslinking protocol and crosslinked with BMB prior to separation by SDS-PAGE and immunoblotting with anti-FtsA.

Table S1. Strains and plasmids used in this study.

**Strain**	**Relevant genotype**	**Source or reference**
DY329	W3110 Δ*lacU169 nadA::Tn10 gal490 pgl*Δ8 λcI*857*Δ*(cro-bioA)*	(1)
XTL970	MG1655 *ΔlacI ΔaraE araFGH*<>spec, *lacY A177C*, *gfp-ftsN*	Xin-tian Li
Top10	Cloning strain	Lab collection
XL1-Blue	Cloning strain	Lab collection
WM1074	MG1655 *ΔlacU169*	Lab collection
WM1115	*ftsA12 (ts)* in WM1074	(2)
WM1125	WM1074 *ftsZ84*(ts)	(3)
WM1657	*ftsA*^{R286W} Δ*zipA::aph* (Δ*zipA::kan)*	(2)
WM2406	pBAD18-FtsL in DY329 recombineering strain	This study
WM2409	WM2406 Δ*ftsL::kan* by recombineering; arabinose dependent	This study
WM2991	W3110 *zipA1*(ts)	(4)
WM4566	WM1074 *ftsA*^{o} + pDSW210-FLAG-FtsA^{G50E}	Lab collection
WM4567	WM1074 *ftsA*^{o} + pDSW210-FLAG-FtsA^{S195P}	Lab collection
WM4572	WM1074 *ftsA*^{o} + pDSW210-FLAG-FtsA^{R286W}	Lab collection
WM4661	WM1074 *leuA::Tn10 ftsQ1*(ts)	(5)
WM4952	MT78 (*leuA::*Tn*10 ftsA*^{o}, *zapA*-GFP, pSC101(ts)-P$_{R}$-*ftsA*	(6)
WM4953	MT79 (MT78 with *ftsL** allele E88K)	(6)
WM7148	pWM7049 (pDSW210FLAG-FtsA^{Q155C}) in *ftsQ1* (WM4661)	this study
WM7149	pWM7049 (pDSW210FLAG-FtsA^{Q155C}) in *zipA1* (WM5337)	this study
WM7150	WM1074 (pDSW210Flag-FtsA^{Q155C}) x P1(*ftsA*^{o} *leuA::*Tn*10*	this study
WM7193	WM1074 (pDSW210Flag-FtsA^{S407C}) x P1(*ftsA*^{o} *leuA::*Tn*10*	this study
WM7237	WM4953 + pDSW210-FtsA$^{M96E\ R153D}$	this study
WM7305	WM1074 + pSEB440-FtsA$^{M96E\ R153D}$	this study
WM7317	WM7305 x P1 (*leuA::*Tn*10 ftsL* ftsA*^{o}*)*	this study
WM7412	WM1125 + pSEB440-FtsA x P1(*ftsA*^{o} *leuA::*Tn*10*)	this study
WM7417	WM4952 + pDSW210-FLAG-FtsA$^{G50E\ Q155C}$	this study
WM7418	WM4952 + pDSW210-FLAG-FtsA$^{G50E\ S407C}$	this study
WM7419	WM4952 + pDSW210-FtsA$^{M96E\ R153D\ Q155C}$	this study
WM7420	WM4952 + pDSW210-FtsA$^{M96E\ R153D\ S407C}$	this study
WM7421	WM4952+ pDSW210-FLAG-FtsA$^{R286W\ Q155C}$	this study
WM7422	WM4952 + pDSW210-FLAG-FtsA$^{R286W\ S407C}$	this study
WM7423	WM4952+ pDSW210-FLAG-FtsA$^{R286W\ Q155C\ S407C}$	this study
WM7451	WM4952 + pDSW210-FtsA$^{M96E\ R153D}$	this study
WM7452	WM4952 + pDSW210-FtsA$^{M96E\ R153D\ G50E}$	this study
WM7453	WM4952 + pDSW210-FtsA$^{M96E\ R153D\ R286W}$	this study

WM7454	WM4953 + pDSW210-FtsA$^{M96E\ R153D\ G50E}$	this study
WM7455	WM4953 + pDSW210-FtsA$^{M96E\ R153D\ R286W}$	this study
WM7462	MG1655 *ΔhsdR ΔendA ΔrecA* (SN1187) *in vivo* cloning strain	(7)
WM7518	pSEB440-FtsA$^{M96E\ R153D}$ in XTL970 x P1 (*leuA::*Tn*10 ftsL* ftsA^{o})*	this study
WM7519	pSEB440-FtsA^{R260W} in XTL970 x P1 (*leuA::*Tn*10 ftsL* ftsA^{o})*	this study
WM7522	WM4952 + pSEB440-FtsA$^{M96E\ R153D\ G50E}$	this study
WM7539	WM4952 + pSEB440-FtsA$^{M96E\ R153D}$ + pBAD18-FtsL**	this study
WM7540	WM4952 + pSEB440-FtsA$^{M96E\ R153D}$ + pDSW210-FtsW*	this study
WM7547	WM1125 + pDSW210-FLAG-FtsA^{Q155C} x P1(*ftsA^{o} leuA::*Tn*10*	this study
WM7550	WM4952 + pDSW210-FLAG-FtsA^{Q155C} (pWM7049)	this study
WM7551	WM4953 + pDSW210-FLAG-FtsA^{Q155C} (pWM7049)	this study
WM7593	WM7518 + pBAD18-FtsL**	this study
WM7594	WM7593 Δ*ftsL::kan* (x P1 WM2409)	this study
WM7599	WM4952 + pSEB440-FtsA$^{M96E\ R153D}$ + pDSW210-FtsA*	this study
WM7600	WM4952 + pSEB440-FtsA$^{M96E\ R153D}$ + pBAD18-FtsL	this study
WM7604	WM4953 + pSEB440	this study
WM7605	WM4953 + pSEB440- FtsA$^{M96E\ R153D}$	this study
WM7606	WM4953 + pSEB440- FtsA$^{M96E\ R153D\ G50E}$	this study
WM7607	WM4953 + pSEB440- FtsA$^{M96E\ R153D\ R286W}$	this study
WM7612	WM1125 + pDSW210-FLAG-FtsA^{D123C}	this study
**Plasmid**	**Description**	**Source or reference**
pUC-GW-Kan	Cloning vector	Azenta Corp.
pDSW210	*colE1* plasmid with weakened P*trc* promoter, Amp^{R}	(8)
pKG110	pACYC184 derivative containing the *nahG* promoter, Cm^{R}	(5)
pSEB439	pACYC184 derivative with a Ptrc promoter	(9)
pSEB440	pSEB439 expressing *ftsA*	(9)
pWM2785	pDSW210-FLAG-FtsA	(10)
pBAD18	*colE1* plasmid with *araBAD* promoter	(11)
pBAD18-FtsL	pBAD18 expressing *ftsL*, arabinose control (pLD45)	(12)
pBAD18-FtsL**	pBAD18 expressing *ftsL*$^{E88K\ G92D}$	this study
pDSW210-FtsW*	pDSW210-FtsW^{E289G}	this study
pWM5732	pDSW210-FLAG-FtsA^{R286W}	this study
pWM5733	pDSW210-FLAG-FtsA^{G50E}	this study
pWM6246	pDSW210-FtsA-msfGFP^{sw}	(13)
pWM7049	pDSW210-FLAG-FtsA^{Q155C}	this study
pWM7181	pDSW210-FLAG-FtsA^{S407C}	this study
pWM7159	pUC-GW-Kan-*ftsA* internal fragment encoding M96E R153D	this study
pWM7215	pSEB440- FtsA$^{M96E\ R153D}$	this study

pWM7235	pDSW210-FLAG-FtsA$^{G50E\ Q155C}$	this study
pWM7236	pDSW210-FLAG-FtsA$^{G50E\ S407C}$	this study
pWM7237	pDSW210-FtsA$^{M96E\ R153D}$	this study
pWM7239	pDSW210-FLAG-FtsA$^{R286W\ Q155C}$	this study
pWM7240	pDSW210-FLAG-FtsA$^{R286W\ S407C}$	this study
pWM7415	pDSW210-FLAG-FtsA$^{M96E\ R153D\ Q155C}$	this study
pWM7423	pDSW210-FLAG-FtsA$^{Q155C\ R286W\ S407C}$	this study
pWM7496	pSEB440- FtsA$^{M96E\ R153D\ R286W}$	this study
pWM7522	pSEB440- FtsA$^{M96E\ R153D\ G50E}$	this study
pWM7612	pDSW210-FLAG-FtsA^{D123C}	this study
pWM7613	pDSW210-FLAG-FtsA$^{M71A\ Q155C}$	this study
pWM7614	pDSW210-FLAG-FtsA$^{M71A\ R286W\ Q155C}$	this study

Table S2. Primers used in this study.

Primer #	5'-3' nucleotide sequence
712 ΔftsL-f	TGCAGAGAGGACGAATGCATGATCAGCAGAGTGACAGAAGGCCACGTTGTGTCTC
713 ΔftsL-r	GCGTTTTCGCCGCTGCTTTCATGCGTCGCGTTTATCCTTAGAAAAACTCATCGAGC
906	CATTGACCAGGCAGAATTGGCCGCAGATTGTCAGATCTC
907	GAGATCTGACAATCTGCGGCCAATTCTGCCTGGTCAATG
2202	GGATAAAGGCGAAGTGAACGACCTCGAATC
2203	ATACCACGCGACGGGCAG
2204	TCGTCCGCCATGGAGTCTGCAAC
2205	CCACCTACGCTCGGCACTTC
2564	CGTGCGGATGTGTGCAAAAGTGCACC
2565	CCCGAAAGTCCTACCGGA
2580	GGTAGTAGGACTGGAGATTGGTACC
2581	CTCCTGAGCATAATCCGTTAAACC
2634	ATCAGTTGGCTGCTGGATCAAGCG
2635	GCTGTAACACGTTTTTCTAC
2659	ATATGGCGAAAAACATCGTCAA
2660	TGCCATCAATTCTGCCTGGTC
2661	CATTGACCAGGCAGAATTGATGGCAGATTGTCAGATCTCTTCGG
2662	GCTTTGACGATGTTTTTCGCCATATCGTTGTGACATGTGATCAGG
2735	GACTTTCGGGCGTCGATATGTGTGCAAAAGTGCACCTGATCAC
2736	GTGATCAGGTGCACTTTTGCACACATATCGACGCCCGAAAGTC
2739	GTTACAGCATCAGTTGGCTGCTGGATCAAGCGACTC
2740	GAGTCGCTTGATCCAGCAGCCAACTGATGCTGTAAC
2814	TTTTTATGAGGCCGCATGCA
2815	TGTTGTTCTGCCTGTGCCTATTCCA
2877	GGCTGTTTTGGCGGATGAGAGAAG
2878	GGCTGTTTTGGCGGATGAGAGAAG
2879	GGATCCTCTAGAATGATCAAGGCGATGCGTTTATCTCTCCCTCGCCTG
2880	CTTCTCTCATCCGCCAAAACAGCCCTGCAGTCATCGTGAACCTCGTAC
2892	CCATACCCGTTTTTTTGGGCTAGCGAATTCCGTATTGCAGAGAGGACGAATG
2893	CATCCGCCAAAACAGCCAAGCTTGCATACTCTAGAGCATGCGTCGCGTTTATCC
2894	ATGCAAGCTTGGCTGTTTTGGCGGATG
2895	GAATTCGCTAGCCCAAAAAAACGGGTATGG
2931	GCGAAATCGGTGCGTGTGCGCTGTGAGCATCGTGTGCTGCATGTG
2932	CACATGCAGCACACGATGCTCACAGCGCACACGCACCGATTTCGC

REFERENCES

1. Thomason LC, Costantino N, Li X, Court DL. 2023. Recombineering: Genetic engineering in *Escherichia coli* using homologous recombination. 2. Curr Protoc 3:e656.

2. Geissler B, Elraheb D, Margolin W. 2003. A gain of function mutation in *ftsA* bypasses the requirement for the essential cell division gene *zipA* in *Escherichia coli*. Proc Natl Acad Sci USA 100:4197–4202.

3. Geissler B, Shiomi D, Margolin W. 2007. The *ftsA** gain-of-function allele of *Escherichia coli* and its effects on the stability and dynamics of the Z ring. Pt 3. Microbiology 153:814–825.

4. Pichoff S, Lutkenhaus J. 2002. Unique and overlapping roles for ZipA and FtsA in septal ring assembly in *Escherichia coli*. EMBO J 21:685-693.

5. Schoenemann KM, Krupka M, Rowlett VW, Distelhorst SL, Hu B, Margolin W. 2018. Gain-of-function variants of FtsA form diverse oligomeric structures on lipids and enhance FtsZ protofilament bundling. Mol Microbiol 109:676–693.

6. Tsang MJ, Bernhardt TG. 2015. A role for the FtsQLB complex in cytokinetic ring activation revealed by an *ftsL* allele that accelerates division. Mol Microbiol 95:925–944.

7. Nozaki S, Niki H. 2019. Exonuclease III (XthA) enforces in vivo DNA cloning of *Escherichia coli* to create cohesive ends. 5. J Bacteriol 201.

8. Weiss DS, Chen JC, Ghigo JM, Boyd D, Beckwith J. 1999. Localization of FtsI (PBP3) to the septal ring requires its membrane anchor, the Z ring, FtsA, FtsQ, and FtsL. J Bacteriol 181:508–520.

9. Pichoff S, Du S, Lutkenhaus J. 2018. Disruption of divisome assembly rescued by FtsN-FtsA interaction in *Escherichia coli.* Proc Natl Acad Sci U S A 115:E6855–E6862.

10. Shiomi D, Margolin W. 2007. Dimerization or oligomerization of the actin-like FtsA protein enhances the integrity of the cytokinetic Z ring. 6. Mol Microbiol 66:1396–1415.

11. Guzman LM, Belin D, Carson MJ, Beckwith J. 1995. Tight regulation, modulation, and high-level expression by vectors containing the arabinose PBAD promoter. 14. J Bacteriol 177:4121–4130.

12. Guzman LM, Weiss DS, Beckwith J. 1997. Domain-swapping analysis of FtsI, FtsL, and FtsQ, bitopic membrane proteins essential for cell division in *Escherichia coli*. J Bacteriol 179:5094–5103.

13. Cameron TA, Margolin W. 2023. Construction and characterization of functional FtsA sandwich fusions for studies of FtsA localization and dynamics during *Escherichia coli* cell division. J Bacteriol 205:e0037322.
